# A MEMS Ultra-Wideband (UWB) Power Sensor with a Fe-Co-B Core Planar Inductor and a Vibrating Diaphragm Capacitor

**DOI:** 10.3390/s21113858

**Published:** 2021-06-03

**Authors:** Sujitha Vejella, Sazzadur Chowdhury

**Affiliations:** Electrical and Computer Engineering Department, University of Windsor, Windsor, ON N9B 3P4, Canada; vejellasujitha@gmail.com

**Keywords:** MEMS, UWB, power sensor, planar inductor, vibrating diaphragm capacitor

## Abstract

The design of a microelectromechanical systems (MEMS) ultra-wideband (UWB) RMS power sensor is presented. The sensor incorporates a microfabricated Fe-Co-B core planar inductor and a microfabricated vibrating diaphragm variable capacitor on adhesively bonded glass wafers in a footprint area of 970 × 970 µm^2^ to operate in the 3.1–10.6 GHz UWB frequency range. When exposed to a far-field UWB electromagnetic radiation, the planar inductor acts as a loop antenna to generate a frequency-independent voltage across the MEMS capacitor. The voltage generates a coulombic attraction force between the diaphragm and backplate that deforms the diaphragm to change the capacitance. The frequency-independent capacitance change is sensed using a transimpedance amplifier to generate an output voltage. The sensor exhibits a linear capacitance change induced voltage relation and a calculated sensitivity of 4.5 aF/0.8 µA/m. The sensor can be used as a standalone UWB power sensor or as a 2D array for microwave-based biomedical diagnostic imaging applications or for non-contact material characterization. The device can easily be tailored for power sensing in other application areas such as, 5G, WiFi, and Internet-of-Things (IoT). The foreseen fabrication technique can rely on standard readily available microfabrication techniques.

## 1. Introduction

Ultra-wideband (UWB) transmission and reception of electromagnetic waves open up a new horizon of applications in the areas of communications and measurement systems, medical diagnostic imaging, patient monitoring, material characterization, and storage tank measurements [1,2]. A UWB device is defined to have a fractional bandwidth greater than 20% within their −10 dB fractional bandwidth in the 3.1–10.6 GHz frequency band [1]. The transmitted short duration pulses in the range of 100s of picoseconds to several nanoseconds duration have a lower power spectral density compared to a narrowband system. These short-duration non-sinusoidal pulses change their waveform and power spectral density as they interact with different dielectric boundaries within a medium during propagation. Thus, a UWB signal reflected from a target medium inherits a unique signature of the interacting target [2,3,4]. A high performance MEMS UWB power sensor can be designed to sense the power of the returned signal for use with an appropriate signal processor to extract information on the target medium.

Experimental evidence presented in [5] shows that a microwave returning from several superimposed yet distinct layers of different dielectric materials when transformed into the time domain indicates the exact depth at which the layer permittivity changes and reveals the propagation characteristics of each traversed layer. As a result, one can ‘see into’ the depths of the stacked materials and the information can be displayed in a graphical format as a power or voltage map.

Such maps can provide valuable information about an otherwise optically opaque medium and its internal composition and shape characterized by dielectric boundaries and can be used for medical and non-destructive evaluation [2,3,6] to detect and identify small low contrast objects.

Significant research is in progress to realize radio frequency (RF) power sensors or detectors operating in narrow band, broadband, and UWB frequency spectrum. Almost all of the reported microelectronics-based RF power-sensing schemes depend on the nonlinear characteristics of CMOS [7], BJT [8], Schottky diodes [9], thermistor [9], thermocouple [9], and current-mode computational circuits [10]. A MEMS capacitive bridge-beam type power sensor operating in the 100 kHz–4 GHz range has been reported in [11]. To the best knowledge of the authors, no MEMS-based UWB power sensor has been reported so far.

The broadband power detection scheme reported in [7] exploits the nonlinearity of a MOSFET channel resistance to generate an average current that is proportional to the input RF power at the drain terminal. A transimpedance amplifier along with a logarithmic amplifier is used to generate a differential voltage from the average current. The device was realized in a 0.18 µm CMOS process and needs gain control to compensate for temperature variation. The narrowband RF power detection schemes reported in [12] need external resonant cavities to couple microwave energies to modulate the drain voltage of a MOSFET. The technique is efficient; however, the requirements for external discrete components and cavities are a major drawback for on-chip integration. A zero-bias JFET-based RF power detection method in the 2.45 GHz has been presented in [13]. In [14], a MOSFET-based power detector for a UWB transmitter has been reported where the square law characteristic of the drain current of a MOSFET in the strong inversion region was used for RMS power detection.

In a discussion on different on-chip power detection schemes presented in [15], the authors mentioned that CMOS incompatible fabrication processes of the Schottky diodes and BJTs were obstacles to frequency-independent on-chip integrated RF power detection, and Joules-heating-based power detection required special packaging. The authors in [15] presented the design of a short channel CMOS-based UWB power detector that has a large dynamic range when used with an additional on-chip matching network. In [10], it was mentioned that “though the peak detection of RF power is suitable for constant-envelope or low peak-to-average ratio modulated signals [16], RMS detection is preferred for high peak-to-average ratio modulated signals due to better accuracy.” In [17], a CMOS-based wideband power detector with high temperature stability within −55 °C to 125 °C and low process-dependent variations was presented; however, the detector’s working frequency range is rather low, from 70 MHz to 1 GHz. The authors in [18] reported that the short channel characteristics of deep submicron technology CMOS, which are also process dependent, cause a deviation of the CMOS I-V characteristics from the square-law domain to result in lower detection sensitivity. To overcome these limitations, a large resistive matching network was used in [18] to realize a CMOS-based RF power detector with a high bandwidth up to 110 GHz. A distributed transmission line like a diode-based network has been implemented in [19] to realize a UWB energy harvester in the 1–11 GHz range. In the design, the authors used a cascade of five commercially available Schottky diodes to distribute the incident RF power among the diode network and a common load resistor. Though the power consumption is low, the device footprint is significantly high, 15 mm × 15 mm, and thus is not suitable for integration in the target applications. 

In this context, this paper presents the design of a MEMS UWB power sensor that generates a voltage corresponding to the magnetic field intensity (amperes/meter) of an incident UWB signal. The sensor can be used as a standalone device. Alternatively, a 2D array of the sensors can be microfabricated to generate a 2D voltage or power map of an incident UWB wavefront carrying the signature of the probed medium (target). Successive 2D maps registered in a regular time interval can be used to create a 3D tomographic map of the target.

One major advantage of the developed sensor over the state-of-the-art RF power detectors as discussed above is that the proposed sensing scheme does not need any circuit modifications for characteristic impedance matching to realize a large bandwidth. While some other detectors rely on an external antenna for the RF signal input, the developed sensor incorporates its own integrated antenna within the same footprint. Furthermore, MEMS capacitive devices are well known to be intrinsically less sensitive to temperature variations with Brownian motion being the main contributor to noise [20,21]. The design of the developed sensor exploits this capability and expands it further by using high-quality HPFS™ glass substrates from Corning™ [22] with a highly stable low coefficient of thermal expansion (CTE), highly stable low dielectric constant, low ionic contamination due to alkali-free manufacturing, and low loss tangent to offer a better signal-to-noise ratio and improved sensitivity. Additionally, the developed device can be batch fabricated at a low cost and integrated with conventional microelectronic-based processing and power units using 3D heterogeneous integration methods to realize a complete system-in-package (SIP).

Another positive feature of the developed sensor that makes it unique compared to other state-of-the-art MEMS or non-MEMS, broadband or UWB RF power detectors is that the developed sensor relies on the deposition and processing of magnetic materials such as Fe-Co-B. Additionally, unlike a microelectronic solid-state RF power detector or a bridge-beam type MEMS device, the operation of the developed sensor depends on the mechanical aspects of a thin vibrational membrane. However, as the operating frequency of the device is well above the fundamental mechanical resonant frequency of the membrane, the mechanical inertia of the membrane highly degrades any high-frequency vibration. Thus, the signal does not modulate the sensor’s capacitance at microwave frequencies, but the RMS value of the signal influences the capacitance to achieve a highly stable dynamic range for the entire target UWB spectrum.

The rest of the paper is organized in the following manner: Section 2 describes the principle of operation; Section 3 presents the theoretical modeling; Section 4 presents the sensor design, including materials and methods; Section 5 provides the results; Section 6 provides a discussion; and Section 7 makes concluding remarks.

## 2. Principle of Operation

**Figure 1:**
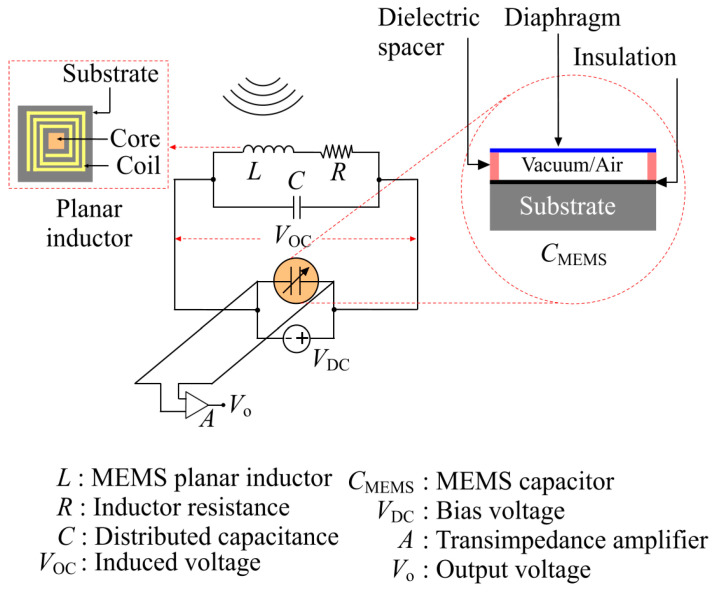
Architecture of the MEMS UWB power sensor. The sensor incorporates a microfabricated Fe-Co-B core planar inductor and a microfabricated vibrating diaphragm variable capacitor. The capacitor generates a frequency-independent output voltage across the transimpedance amplifier in response to an induced voltage across its electrodes by the planar inductor due to an incident UWB signal.

The architecture of the MEMS UWB power sensor is presented in Figure 1. The sensor is built with a microfabricated square cross-section planar inductor L connected in parallel to a microfabricated MEMS variable capacitor $C_{\mathrm{MEMS}}.$

The capacitor $C_{\mathrm{MEMS}}$ is constructed with a deformable square diaphragm (top electrode), which is separated from a fixed backplate (bottom electrode) by a dielectric spacer enclosing a vacuum or an airgap. When the sensor is exposed to an electromagnetic radiation field in the target UWB frequency spectrum, the inductor acts as a loop antenna to induce an alternating voltage across its terminals, following Faraday’s law of electromagnetic induction.

**Figure 2:**
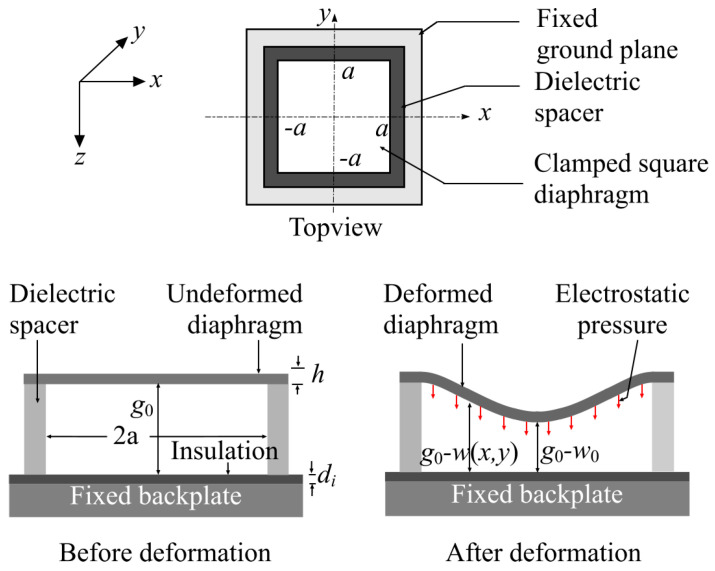
Operation of the MEMS deformable diaphragm variable capacitor.

This induced voltage $V_{\mathrm{OC}}$ superposed by a bias voltage $V_{\mathrm{DC}}$ accumulates opposite charges on the capacitor electrodes to generate a coulombic attraction force between the diaphragm and the backplate. As the backplate (bottom electrode) is rigidly fixed and the diaphragm (top electrode) is thin with rigidly clamped edges, this force deforms the diaphragm as shown in Figure 2. In Figure 2, $g_{0}$ is the zero-force gap, a is half of the diaphragm side-length, h is the diaphragm thickness, $d_{i}$ is the thickness of the bottom insulation layer, and $w_{0}$ is the deflection of the diaphragm center. The deformation modifies the gap between the diaphragm and the backplate of the capacitor to affect a change in capacitance. This capacitance change is sensed using a transimpedance amplifier along with a DC bias to generate an output voltage $V_{o}$ as shown in Figure 1.

If the frequency of the induced AC voltage across the MEMS capacitor is comparable to the fundamental mechanical resonant frequency of the diaphragm, the diaphragm vibrates at the frequency of the induced voltage. However, if the fundamental mechanical resonant frequency of the diaphragm is much lower than the frequency of the induced AC voltage, the diaphragm deflection cannot follow the time variation of the resulting oscillating electrostatic force due to inertia and depends only on the RMS magnitude of it [11,23], making the capacitance change independent of the frequency of the induced voltage.

Additionally, as the electrostatic attraction force is proportional to the square of the voltage [9], the diaphragm deflection is also proportional to the square of the voltage. As the power is also proportional to the square of the voltage, the diaphragm deflection becomes a linear function of power, and the corresponding output voltage can be easily calibrated to the magnetic field strength, electric field strength, or power density [24].

## 3. Theoretical Modeling

**Figure 3:**
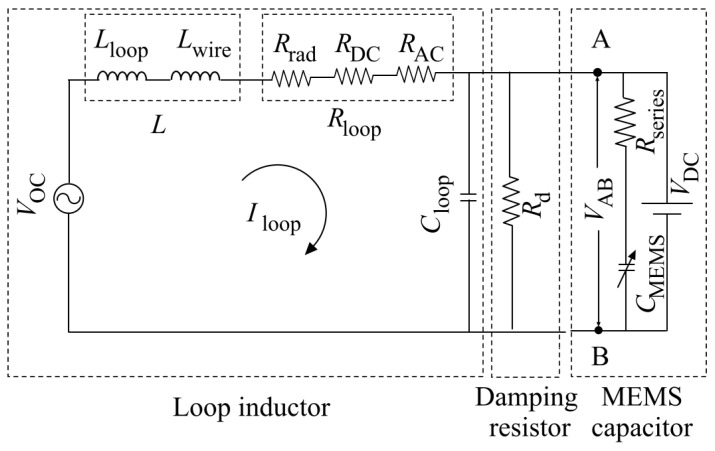
Electrical equivalent circuit of the MEMS UWB power sensor.

An electrical equivalent circuit of the MEMS UWB power sensor is shown in Figure 3. In the Figure, $V_{\mathrm{OC}}$ is the open circuit voltage induced across the inductor, L is the lumped inductance of the square-loop antenna comprising of inductance of the loop $L_{\mathrm{loop}}$ and self-inductance of the conductor

$L_{\mathrm{wire}}.$ The lumped resistance of the loop $R_{\mathrm{loop}}$ is comprised: (1) radiation resistance $R_{\mathrm{rad}}$ corresponding to the loss in the antenna during the transformation of EM energy into electrical energy, (2) loss due to the DC resistance of the conductor $R_{\mathrm{DC}},$ and (3) AC loss due to the skin effect $R_{\mathrm{AC}}.$ In Figure 3, $C_{\mathrm{loop}}$ represents the distributed capacitance of the square loop, and $C_{\mathrm{MEMS}}$ represents the variable capacitance between the deformable diaphragm and the backplate, which is in series with the capacitor self-resistance $R_{\mathrm{series}}$. A damping resistor $R_{d}$ is used between the inductor L and the MEMS variable capacitor $C_{\mathrm{MEMS}}$ to enable a flat frequency response over the target UWB frequency range. These parameters can be calculated following the mathematical models available in [25]. A DC bias voltage $V_{\mathrm{DC}}$ is superimposed on the actuating AC voltage $V_{\mathrm{AB}}$ across the MEMS capacitor to set the static operating point and facilitate capacitive readout.

Assuming a constant current around the loop (small loop approximation) and the loop antenna is far from the source of the incident field; the induced open circuit AC voltage $V_{\mathrm{OC}}$ across the inductor due to an incident time-varying sinusoidal electromagnetic wave with an angular frequency ω can be expressed as [26,27,28]:

(1) $$V_{\mathrm{OC}}=\sqrt{2}V_{\mathrm{RMS}}\cos\omega t$$

where $V_{\mathrm{RMS}}$ is the RMS value of the induced voltage expressed as [27]

(2) $$V_{\mathrm{RMS}}=2\pi \mu_{0}\mu_{r}NA_{\mathrm{loop}}fH_{\mathrm{RMS}}\cos\theta$$

In (2), $H_{\mathrm{RMS}}$ is the RMS value of the magnetic field intensity (A/m) of an incident electromagnetic wave, $\mu_{0}$ is the vacuum permeability (H/m), $\mu_{r}$ is the relative permeability of the medium, N is the number of turns in the loop, $A_{\mathrm{loop}}$ is the area of each turn (m^2^), f is the frequency of the incident wave (Hz), and θ is the angle between the magnetic flux lines and the plane normal to the loop surface. Assuming negligible loss in the loop, the electrostatic force between the diaphragm and the backplate can be calculated following [25]

(3) $$F_{\mathrm{electrostatic}}=\frac{\varepsilon_{0}A\left(V_{\mathrm{DC}}^{2}+V_{\mathrm{RMS}}^{2}+2\sqrt{2}V_{\mathrm{DC}}V_{\mathrm{RMS}}\cos\omega t+V_{\mathrm{RMS}}^{2}\cos2\omega t\right)}{2{\left(\frac{d_{i}}{\varepsilon_{i}}+\left(g_{0}-w_{\mathrm{RMS}}\right)\right)}^{2}}$$

where $\varepsilon_{0}$ is the permittivity of free space, A is the coupling area of the capacitor electrodes, $d_{i}$ is the thicknesses of a bottom insulation layer to prevent device damage at pull-in (Figure 2), $\varepsilon_{i}$ is the relative permittivity of the insulating layer material, and $w_{\mathrm{RMS}}$ is the piston-like RMS deflection of the diaphragm [29,30,31]. The piston-like RMS displacement of the diaphragm $w_{\mathrm{RMS}}$ can be calculated following [29,30,31] as $w_{\mathrm{RMS}}=0.4243w_{0}$, where $w_{0}$ is the deflection of the diaphragm center (Figure 2).

The first term in the numerator on the right-hand side of (3) corresponds to the electrostatic force generated due to the DC bias voltage; the second term corresponds to the electrostatic force due to the RMS voltage of the AC component; and the third and fourth terms correspond to the forces exerted by the voltages oscillating at the frequency equal to and double the frequency of the induced voltage. If the frequency of the induced voltage is well above the mechanical resonant frequency of the diaphragm, the diaphragm vibration cannot follow the frequency of the resulting oscillating electrostatic force. Consequently, the induced voltage does not change the capacitance at UWB frequencies but the RMS value of the induced voltage determines the capacitance change [11,23,25]. Thus, the third and fourth terms in (3) can be neglected to obtain the effective electrostatic force between the capacitor electrodes as

(4) $$F_{\mathrm{electrostatic}}=\frac{\varepsilon_{0}A\left(V_{\mathrm{DC}}^{2}+V_{\mathrm{RMS}}^{2}\right)}{2{\left(\frac{d_{i}}{\varepsilon_{i}}+\left(g_{0}-w_{\mathrm{RMS}}\right)\right)}^{2}}$$

To simplify the load-deflection analysis of the diaphragm, it is assumed that the diaphragm is made of an isotropic homogeneous conducting elastic material with rigidly clamped perfect edge conditions. It is also assumed that the diaphragm is at room temperature and there is no temperature gradient in the system. Furthermore, the electric field between the diaphragm and backplate is assumed to be uniform. Under such assumptions, the center deflection $w_{0}$ of the diaphragm due to the electrostatic force expressed in (4) can be calculated from the following load-deflection relation of a square diaphragm [32]

(5) $$P_{\mathrm{electrostatic}}=\left[C_{r}\frac{\sigma_{0}h}{a^{2}}+C_{b}\frac{12D}{a^{4}}\right]w_{0}+\left[C_{s}f_{s}\left(v\right)\frac{\tilde{E}h}{a^{4}}\right]w_{0}^{3}$$

where $P_{\mathrm{electrostatic}}=F_{\mathrm{electrostatic}}/A.$

In (5), $\tilde{E}$ is the effective Young’s modulus, D is the flexural rigidity of the diaphragm, $\sigma_{0}$ is the residual stress, v is the Poisson ratio, h is the diaphragm thickness, a is half of the diaphragm sidelength (Figure 2), and $C_{r}=3.45,\;C_{b}=4.06,\;\mathrm{and}\;C_{s}=1.994$.

The first term within the first square bracket on the right-hand side of (5) represents a linear stiffness term due to the residual stress $\sigma_{0}$, the second term within the first square bracket on the right-hand side of (5) represents a linear stiffness term due to bending, and the term within the second square bracket on the right-hand side of (5) represents a cubic stiffness term. The presence of the cubic stiffness term in (5) characterizes a nonlinear spring-hardening phenomenon where the stiffness increases nonlinearly with deflection due to in-plane stretching of the diaphragm at large deflections [32].

The flexural rigidity, D, of the diaphragm can be calculated following:

(6) $$D=\frac{Eh^{3}}{12(1-v^{2})}$$

where E is the Young’s modulus. The effective Young’s modulus $\tilde{E}$ of the diaphragm in (5) depends on the Poisson’s ratio v as

(7) $$\tilde{E}=\frac{E}{1-v^{2}}$$

Assuming that the capacitor geometry is realized using a vacuum-based wafer bonding process to realize a vacuum inside the cavity [25], any fluid-damping effect is neglected in the analysis.

The resonant frequency of an air-coupled square diaphragm can be calculated from [33]

(8) $$f_{r}=\sqrt{\frac{1}{\rho }\left(\frac{D\pi^{2}}{b^{4}}+\frac{T}{2b^{2}}\right)}$$

where *T*, *ρ*, and b are the tensile force per unit length ($T=\sigma_{0}h$), the diaphragm mass per unit area, and sidelength of the square diaphragm (b=2a), respectively. The capacitance of the sensor capacitor can be calculated from [32]

(9) $$C_{\mathrm{MEMS}}=C_{0}(1+C_{\mathrm{ff}})$$

where the ideal parallel plate capacitance

(10) $$C_{0}=\frac{\varepsilon_{0}A}{\frac{d_{i}}{\varepsilon_{i}}+\left(g_{0}-w_{\mathrm{RMS}}\right)}$$

and $C_{\mathrm{ff}}$ is the fringing field factor expressed as [21]

(11) $$C_{\mathrm{ff}}=\frac{0.385}{a}\left[\frac{d_{i}}{\varepsilon_{i}}+g_{0}\right]+1.06{\left[\frac{1}{2a}\left(\frac{d_{i}}{\varepsilon_{i}}+g_{0}\right)\right]}^{0.75}$$

For the sake of simplicity, in the analysis it is assumed that the series and parallel parasitic capacitances are negligible.

## 4. Sensor Design and Simulation

Unlike a conventional LC tank circuit, the design challenge is to realize a flat frequency response of the inductor (antenna) in the target UWB frequency range and maximize the electrostatic attraction force between the capacitor plates to maximize the frequency-independent sensitivity for the target UWB spectrum.

### 4.1. Flat Frequency Response for the Antenna Loop

Following Figure 3, the induced phasor-domain current, ${\hat{I}}_{\mathrm{loop}},$ can be expressed as a function of the phasor-domain voltage, $\hat{V}$ as

(12) $${\hat{I}}_{\mathrm{loop}}=\frac{\hat{V}}{(R_{\mathrm{loop}}+jX_{L})+\left(\frac{1}{jX_{C_{\mathrm{loop}}}}+\frac{1}{R_{d}}+\frac{1}{R_{\mathrm{series}}+jX_{C_{\mathrm{MEMS}}}}\right)}$$

To obtain a flat frequency response in the target UWB frequency range, the antenna loop should operate in a short circuit mode [28,34] as a highpass filter comprised of $X_{L}$ and $R_{\mathrm{loop}}$ with a cut-off frequency defined by $f_{u}=R_{\mathrm{loop}}/(2\pi L)$. Running the loop in a short circuit mode, the distributed capacitive reactance $X_{C_{\mathrm{loop}}}$ has no effect on the output, ensuring immunity against parasitic capacitance and less coupling with nearby objects [34]. However, the damping resistor $R_{d}$ in parallel with $C_{\mathrm{loop}}$ creates a lowpass filter with a cut-off frequency $f_{l}=1/(2\pi R_{d}C_{\mathrm{loop}})$ [28]. By making $X_{C_{\;\mathrm{MEMS}}}+R_{\mathrm{series}}>>R_{d}$, the current ${\hat{I}}_{\mathrm{loop}}$ in (8) can be approximated as

(13) $${\hat{I}}_{\mathrm{loop}}\approx \frac{\hat{V}}{R_{\mathrm{loop}}+jX_{L}}$$

As $\hat{V}$ and $X_{L}$ are both functions of frequency, and in the high pass regime, $X_{L}>>R_{\mathrm{loop}}$, ${\hat{I}}_{\mathrm{loop}}$ becomes independent of frequency. Thus, by proper selection of circuit components, it is possible to obtain a flat frequency response in the target UWB frequency range. It is to be noted here that $R_{\mathrm{loop}}$ is not strictly frequency independent due to the “skin effect”. The voltage ${\hat{V}}_{\mathrm{AB}}={\hat{I}}_{\mathrm{loop}}R_{d}$ across the damping resistor is the actuating voltage for the MEMS capacitor.

**Figure 4:**
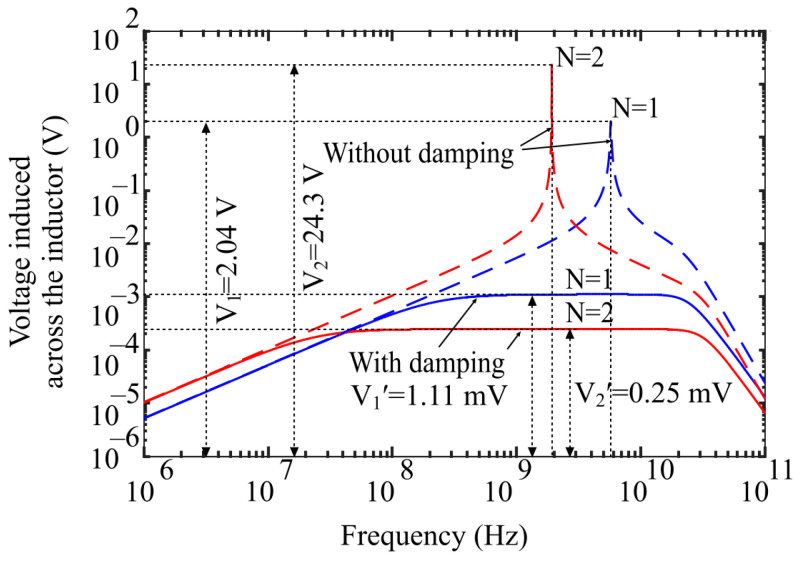
Frequency response of the loop inductor with and without a damping resistance.

However, this wideband characteristic of the resulting circuit decreases the amplitude of the voltage across the sensor capacitor $C_{\mathrm{MEMS}}$ as it is evident from the simulation results in Figure 4. To maximize the sensitivity (ΔC/H), it is necessary to maximize the diaphragm deflection $w_{0}$ by increasing ${\hat{V}}_{\mathrm{AB}}$ while ensuring that ${\hat{V}}_{\mathrm{AB}}$ does not exceed the pull-in limit.

**Figure 5:**
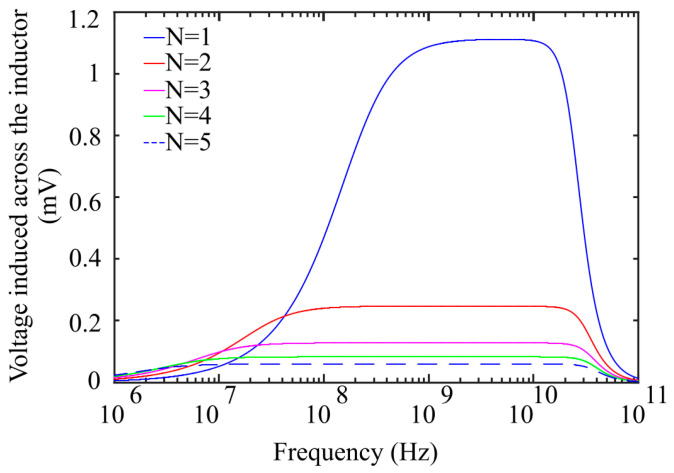
Voltage generated across the loop inductor as a function of number of loop turns N.

As ${\hat{V}}_{\mathrm{AB}}$ is inversely proportional to the number of loop turns N [25], a smaller N results in higher ${\hat{I}}_{\mathrm{loop}}$, contributing to higher ${\hat{V}}_{\mathrm{AB}}$ as shown in Figure 5. Accordingly, a single turn square loop was selected for the proposed design.

**Figure 6:**
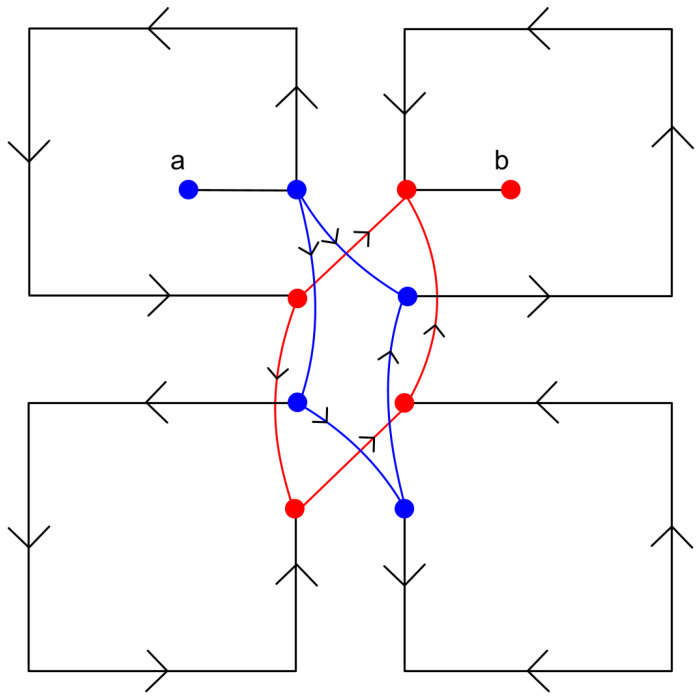
Crossed parallel sub-loop inductors with current flow directions from node a to node b.

As the loop current ${\hat{I}}_{\mathrm{loop}}$ depends on $A_{\mathrm{loop}}/L_{\mathrm{loop}}$ ratio, for a fixed loop area $A_{\mathrm{loop}}$, the current can be increased further to increase ${\hat{V}}_{\mathrm{AB}}$ by decreasing the loop inductance $L_{\mathrm{loop}}.$ An investigation shows that an approach based on crossed parallel sub-loops [35] as shown in Figure 6 can reduce the loop inductance further without affecting the loop area $A_{\mathrm{loop}}$ and the antenna radiation pattern.

**Figure 7:**
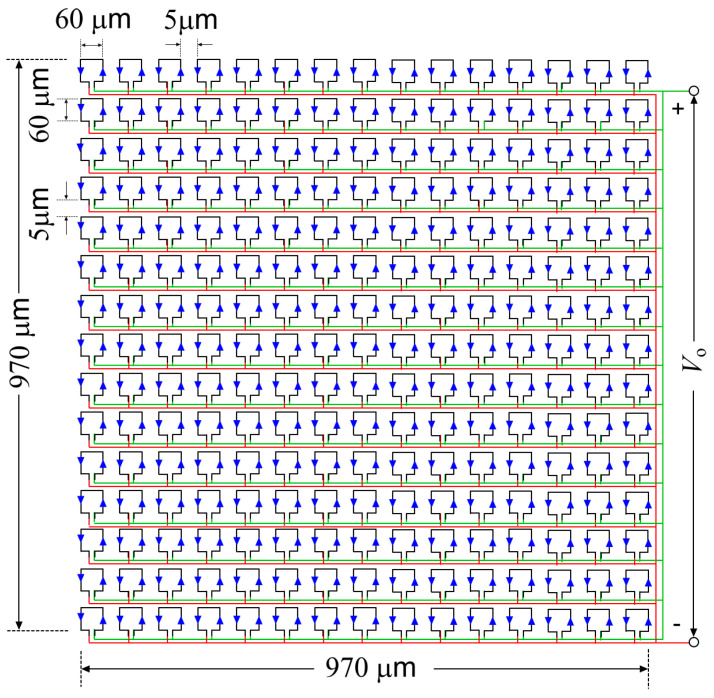
15 × 15 crossed parallel sub-loop inductors.

To realize a crossed parallel sub-loop based inductor, the single loop is divided into several smaller loops keeping the same total area and cross-connecting them as shown in Figure 6, so that the current directions of adjacent arms of the successive inductors oppose each other to reduce the mutual inductance. With this configuration, the effective loop inductance is lowered and the loop current is maximized for the same lateral dimension [14]. Following this approach, simulations of different configurations of crossed parallel sub-loops are conducted in PSpice^®^ by varying the number of sub-loops $\left(n_{s}\right)$, the length of the conductor, and the width of the conductor to determine the final specifications of the loop inductor. The connections among the sub-loops are shown in Figure 7. To minimize $R_{\mathrm{loop}}$, gold was selected as the material for the loop inductor due to its high conductivity and ease of microfabrication.

Following (2), a high permeability core is necessary to achieve a high sensitivity by increasing the induced voltage. However, as the permeability of ferromagnetic materials drops sharply beyond the ferromagnetic resonance frequency $f_{\mathrm{FMR}},$ a ferromagnetic material with stable high permeability and also a high $f_{\mathrm{FMR}}$ beyond the upper limit of the target UWB frequency range is necessary to increase the sensitivity. The ferromagnetic resonance frequency $f_{\mathrm{FMR}}$ depends on the magnetic anisotropy ($H_{k}$) and saturation magnetization ($4\pi M_{s}$) following [36]

(14) $$f_{\mathrm{FMR}}=\gamma {\left(H_{k}M_{s}\right)}^{1/2}/2\pi$$

where γ is the gyromagnetic ratio. Following (14), either $H_{k}$ or $M_{s}$ can be enhanced to increase $f_{\mathrm{FMR}}$ of ferromagnetic materials, such as sputter-deposited Fe-Co-B thinfilms. However, the researchers prefer enhancement of $H_{k}$ compared to the enhancement of $M_{s}$ as the latter is capped at 24.5 kG at room temperature [36,37,38,39,40,41]. In [36], experimental results are presented to confirm that embedding ultra-fine layers of Si/NiFe in 200-nm-thick layers of Fe-Co-B films contribute to large saturation magnetization of 20 kG and high in-plane magnetic anisotropy of 257 Oe. The technique in [36] resulted in a measured ferromagnetic resonance at 6.4 GHz with a stable permeability $\mu^{\prime }$ of at least 70 in the 3.0–6.4 GHz range. The authors in [36] predicted that a theoretical ferromagnetic resonance frequency of 9.4 GHz can be achieved with a magnetic anisotropy of 500 Oe and suggested using multilayer Fe-Co-B films with Si/NiFe intermediate layers to suppress the loss and achieve good permeability at high frequency range. This prediction was experimentally verified to achieve experimental $f_{\mathrm{FMR}}$ of 9.2 GHz ($H_{k}=$ 500 Oe) [40] and 12.96 GHz ($H_{k}=$1498 Oe) [41]. However, [40] and [41] did not provide any permeability values. Based on these experimentally measured $f_{\mathrm{FMR}}$ and permeability values, a relative permeability of 70 is chosen for the Fe-Co-B core to design the inductor with an expectation that the current trend of ongoing research will continue to achieve a permeability value of at least 70 up to the upper limit (10.6 GHz) of the target UWB frequency range in the near future.

### 4.2. MEMS Capacitor Design

The sensitivity of the capacitor can be defined as the change in capacitance (ΔC) with respect to a change in the applied voltage (ΔV). In addition to maximizing ${\hat{V}}_{\mathrm{AB}}$ and a proper selection of $V_{\mathrm{DC}}$, sensitivity can be further optimized using a thin metallic diaphragm and a small gap between the diaphragm and the backplate. To minimize ohmic losses, high conductivity material is preferable for the capacitor electrodes. Based on these considerations, a thin film of gold was selected as the diaphragm material. Equations (3)–(11) can be used to calculate the deflection of the diaphragm center and calculate the capacitance.

To make $X_{C_{\;\mathrm{MEMS}}}>>R_{d}$, $R_{d}$ is determined following the minimum measurable capacitance change calculated following (9)–(11). The geometrical dimensions of the capacitor have been determined to yield a minimum capacitance change resolution of 1 aF that is achievable following [42,43]. At 3.1–10.6 GHz, range this yields a capacitive reactance of approximately 31 MΩ, which is much larger than the selected 3Ω value for the damping resistance $R_{d}$ satisfying the design requirements. 

### 4.3. Material Selection

Corning™ HPFS™ glass substrate was selected for the substrate for the loop antenna due to its low coefficient of thermal expansion (CTE) 31.7 × 10^−7^/°C, low ionic contamination due to alkali-free manufacturing, low loss tangent 0.007@10 GHz, low dielectric constant 5.15@10 GHz, excellent surface roughness (<1.0 nm), and low warping [22,44,45]. Copper is ideally the material of choice as copper-on-glass achieves better conductivity than other conductors [45]. However, due to the risk of copper oxidation and approximately 14.5% lower skin depth compared to gold at the upper limit of the target 3.1–10.6 GHz, gold was selected as the metal layer on Corning™ HPFS glass substrate for the planar inductor. In [46], it was reported that small roughness on the scale of a few atoms on the perfect surface of thinfilm conductors distorts the isotropic Fermi surface sheets, reducing the electrical conductivity significantly, approximately by 30–40%. Thus, smoother thinfilm metals are more conducting than rough thinfilm metals to lower the insertion loss. Additionally, the low dielectric constant of a glass substrate lowers down the parasitic capacitance. Both factors contribute to achieve a high quality factor Q [45]. Thus, the use of gold on HPFS™ glass substrate helps achieve a high Q for the inductor by lowering the insertion loss and parasitic capacitance.

Gold was also selected to realize the diaphragm of the MEMS capacitor. Due to excellent electrical and mechanical properties of benzocyclobutene (BCB), a low κ (2.65) polymer from Dow™ Chemical Company was selected as the bottom insulation layer to avoid a short circuit in the event of a pull-in [47]. BCB was also used as a low dielectric constant spacer between the capacitor electrodes to lower the parasitic capacitance and minimize dielectric charging, thus enhancing the electromechanical energy transduction efficiency. Finally, BCB was used as a low-temperature adhesive bonding agent to realize the vacuum capacitor cavity (gap) by adhesively bonding two partially processed glass wafers to complete the device. 

## 5. Results

The final design specifications of the MEMS UWB power sensor are provided in Table 1 below.

The detailed specifications for the sensor inductor determined following the design method as mentioned before are provided in Table 2.

To attain flat frequency response of the sensor in the operating UWB range, the sensor capacitance range was decided to be in the range of 1–2 pF. Following (17), the resistance $R_{d}$ was selected to be 3Ω. As the value of $R_{d}$ was selected, the voltage across the resistor totally depended on the induced current. Taking these considerations and fabrication issues [25] into account, an optimal set of specifications, as shown in Table 3 [25], were determined for the MEMS capacitor using the material properties as listed in Table 4 [25].

### 5.1. Resonant Frequency Simulation

**Figure 8:**
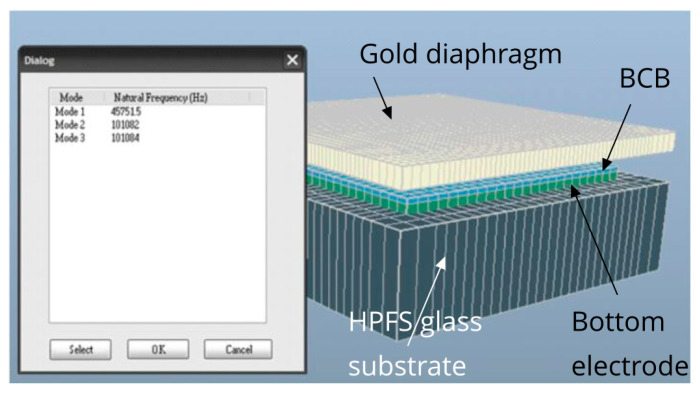
Sensor capacitor 3D meshed model in IntelliSuite™. The inset image shows the first three modes of resonant frequencies.

For the designed diaphragm geometry, the fundamental resonant frequency of the sensor capacitor diaphragm was determined from a 3D finite element analysis (FEA) simulation using IntelliSuite™ [48] as 45.75 kHz. The simulation model is shown in Figure 8. Fixed boundary conditions were applied to the sides of the diaphragm. The inset in Figure 8 shows the first three modes of the resonant frequencies. As the target 3.1–10.6 GHz UWB spectrum was far away from the fundamental resonant frequency of the diaphragm (45.75 kHz), only the RMS value of the incident UWB signal contributed to the electrostatic force in Equation (4) [11].

### 5.2. Electromechanical 3D FEA Simulation of Pull-in Voltage of the MEMS Capacitor

The static pull-in voltage for the MEMS capacitor geometry was determined from a thermo-electro-mechanical relaxation-based 3D finite element analysis (FEA) in IntelliSuite™ [48]. To set up the simulation, fixed boundary conditions were applied to all four sides of the top diaphragm while free boundary conditions were applied to the top and bottom faces of the top diaphragm. For the bottom electrode, fixed boundary conditions were applied to all six faces. As mentioned in [48], the thermo-electro-mechanical relaxation method repeatedly used a boundary element solver to calculate and update capacitance and charge information and a finite element solver to calculate and update mechanical deformations until a target convergence was achieved. Triangular boundary elements were chosen for electrostatic analysis while hexahedral 20-node brick elements were chosen for mechanical analysis to result in a smaller computational model with improved numerical accuracy [48]. The use of 20-node brick elements also reduced the need for fine discretization through the thickness of the model as necessary for typical 8-node brick element based-simulations [48].

**Figure 9:**
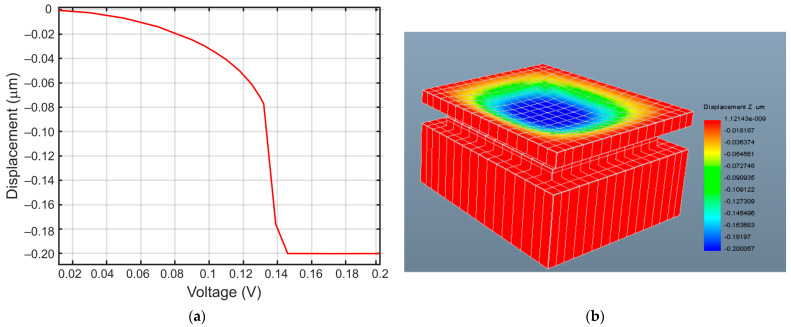
Pull-in voltage simulation of the sensor capacitor in IntelliSuite™. (**a**) IntelliSuite™ 3D electromechanical FEA result showing the displacement of the diaphragm center due to the nonlinear electrostatic force following (4) as a function of bias voltage; (**b**) 3D FEA model of the sensor capacitor geometry in IntelliSuite™ design environment showing the collapsed diaphragm after pull-in.

The resulting transfer curve showing the nonlinear displacement of the diaphragm center as a function of bias voltage is shown in Figure 9a. The corresponding 3D FEA model of the capacitor sensor geometry is shown in Figure 9b, which shows the collapsed diaphragm after pull-in.

### 5.3. Dynamic Analysis

**Figure 10:**
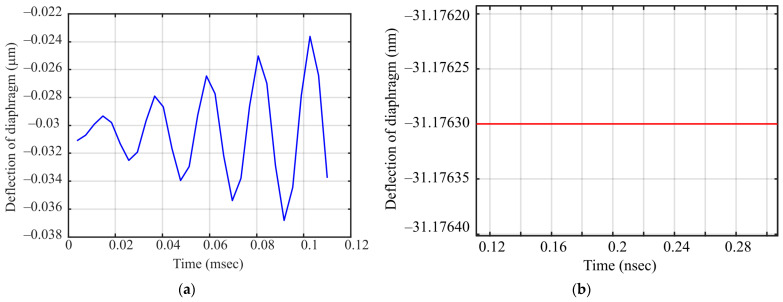
(**a**) 3D FEA transient analysis results showing the diaphragm deflection at different frequencies. (**a**) Diaphragm deflection for 1 mV AC voltage at 45.75 KHz (mode 1); (**b**) diaphragm deflection for 1 mV amplitude AC voltage at 5.1 GHz.

As the fundamental resonant frequency of 45.75 kHz (Figure 8) of the diaphragm was much lower than the target 3.1–10.6 GHz UWB frequency range, only the RMS value of the induced voltage across the capacitor contributed to the electrostatic force, causing a deflection of the diaphragm. To verify the fact, a 3D FEA-based transient analysis was conducted to determine the diaphragm deflections for 1 mV AC voltage at 45.75 KHz and for 1 mV AC voltage at 5.1 GHz. The resulting diaphragm deflections as a function of time are shown in Figure 10. Figure 10a shows the deflection of the diaphragm at 45.75 kHz AC voltage actuation, while Figure 10b shows the deflection at 5.1 GHz AC voltage actuation. As it can be seen, for the 5.1 GHz AC voltage actuation, the deflection was constant at approximately 31.17 nm and no oscillation of the diaphragm with respect to the actuation frequency can be observed. This observation is consistent with (4) that predicts that for actuation at frequencies much higher than the resonant frequency of the diaphragm, the diaphragm vibration cannot follow the oscillating frequency due to inertia and RMS value of the electrostatic force that contributes to deflection. A much larger amplitude oscillation of the diaphragm near fundamental resonant frequency is visible in Figure 10a.

### 5.4. Sensitivity Calculation

**Figure 11:**
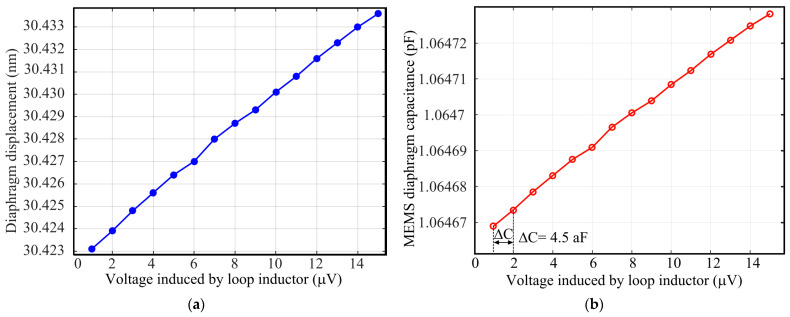
(**a**) 3D FEA simulated diaphragm deflection; (**b**) 3D FEA simulated capacitance change due to the induced voltage.

Figure 11a shows 3D FEA-simulated deformation of the capacitor diaphragm for a parametric voltage sweep using IntelliSuite™, and Figure 11b shows the corresponding capacitance of the MEMS capacitor. Figure 11a reveals that the center deflection of the diaphragm is linear with increasing actuation voltage, and a deflection variation of ~0.8 pm is observed for every 1 µV change in the actuation voltage (loop inductor induced voltage). This deflection variation translates to a mechanical sensitivity of 0.8 pm/µV. The corresponding capacitance change is 4.5 aF/µV, as can be seen in Figure 11b.

The output voltage from the cross-parallel loop inductor array was calculated using MATLAB^®^ and OrCAD^®^ PSpice^®^ as 1 µV due to an incident magnetic field intensity of 0.8 µA/m. The sensitivity of the MEMS sensor can thus be derived as 4.5 aF/0.8 µA/m.

## 6. Discussion

The developed sensor enables a non-contact method for RF RMS power sensing from a far field source or reflecting boundary in the 3.1–10.6 GHz UWB frequency range with a calculated sensitivity of 4.5 aF/0.8 µA/m. One major advantage of the designed sensor is that it does not need any additional compensating circuit elements to retune the characteristic impedance to realize a broadband capability that degrades the loss parameters [8]. Additionally, the use of thermally stable alkali-free low-loss tangent ultra-planar (<10 nm) low dielectric constant glass substrate is poised to enhance the signal-to-noise ratio significantly to enable improved detection, contrast, and resolution in a wide area of applications [45,49]. As the deformation of the gold diaphragm is smaller than its thickness and considering the relatively low Young’s modulus of gold, the generated stress is expected to be much smaller than the yield stress of gold to ensure reliable operation over a long period of time. Due to lower ionic contamination of BCB and low operating voltage, the electric field across the BCB dielectric spacer is expected to be much smaller to cause a breakdown of BCB or to create dielectric polarization of BCB. The leakage current is expected to be negligible.

One of the limitations of the proposed sensor is that a sophisticated capacitive readout circuit is necessary to read the small aF scale capacitance change [42,43]. Advanced materials, geometric modifications, and advanced capacitive readout circuits with a lower noise floor are under investigation to increase the capacitance change to achieve higher sensitivity.

As the generated voltage output from the MEMS capacitor is frequency independent and the planar inductor loop current is also frequency independent, the device can be tailored as a non-contact RF power sensing device for other frequencies of interest, e.g., to use it for 5G applications, WiFi (2.5 GHz), Bluetooth, and to monitor the radiation level in high population areas.

The device can be batch fabricated at a lower cost and can also become a valuable tool in non-contact evaluation of medical conditions or material characterization of an otherwise optically opaque medium with internal dielectric boundaries.

Extensive research is in progress to realize high Q on-chip inductors by fabricating magnetic softcore materials with high permeability at high frequencies. Once ferromagnetic thinfilms with high permeability values in the range of 1000 or higher are available, the device inductor can become smaller to reduce the sensor form factor.

The device was designed using industry standard MEMS design tool IntelliSuite™. The foreseen fabrication technique can rely on standard readily available microfabrication techniques. The development of a fabrication process to fabricate the sensor using an adhesive wafer bonding technique is in progress using Intellifab™, the virtual cleanroom module of the IntelliSuite™ design suite. Once the fabrication process development is completed and necessary funding is available, the device will be fabricated and tested. As a preliminary task, the authors have already experimentally verified a BCB-based adhesive bonding technique [47].

## 7. Conclusions

The design and simulation results of a MEMS-based UWB power sensor were presented. The 970 × 970 µm^2^ footprint area sensor uses a planar microfabricated inductor and a vibrating diaphragm MEMS capacitor to generate a frequency-independent output voltage over the designated 3.1–10.6 GHz UWB frequency range. The sensor exhibited a calculated sensitivity of 4.5 aF/0.8 µA/m. Static and dynamic 3D thermoelectromechanical finite element analyses were conducted to calculate natural frequencies, pull-in voltage, and frequency response of the MEMS capacitor in the target frequency spectrum, to verify the design. The sensor can be used as a standalone UWB power sensing device. Alternatively, a 2D array of the sensor can be used to generate a 2D voltage or power map of an incident UWB wavefront carrying the signature of a probed medium (target). Successive 2D maps registered in a regular time interval can be used to create a 3D tomographic map of the target. The inherent frequency-independent ultra-wideband response of the sensor can be tailored further for other RF power sensing applications, for example, 5G, WiFi (2.45 GHz), and for Internet-of-Things (IoT) applications.

Unlike other UWB or broadband RF power detectors [7,8,10,12,13,14,15,16,17,18,19], the UWB Fe-Co-B planar antenna is an integral part of the proposed sensor. Such integration reduces the form factor, eliminates any need for post integration using external components, and enables one to batch fabricate the device at a low cost.

The development and simulation of a fabrication method using IntelliFab™ virtual cleanroom is in progress. The method incorporates microfabrication processes and materials that are available in standard microfabrication facilities. The device will be fabricated and tested once the fabrication method development is complete and necessary funding is available.

## Figures and Tables

**Table 1:** MEMS UWB power sensor final specifications.

Parameter	Value	Unit
Sensor footprint area	970 × 970	μm^2^
Inductor area	970 × 970	μm^2^
Capacitor area	150 × 150	μm^2^
UWB frequency range	3.1–10.6	GHz
DC bias	0.1	V
Sensitivity	4.5 aF/0.8 µA	

**Table 2:** Sensor inductor specifications.

Parameter	Value	Unit
Total equivalent loop area	970 × 970	μm^2^
Total inductance, L	58.13	pH
Gap between the sub-loops, $g_{s}$	5	µm
Square-loop sidelength, w	60	µm
Number of turns, N	1	-
Width of the conductor, d	1	µm
Number of sub-loops, $n_{s}$	225 (15 × 15)	-
Thickness of the conductor, $t_{d}$	1	µm
Sub loop inductance, $L_{s}$	13.07	nH
Conductor material	Gold	
Core material	Fe-Co-B	
Substrate	HPFS™	

**Table 3:** MEMS capacitor design specifications [25].

Parameter	Value	Unit
Diaphragm side length, 2a	150	µm
Diaphragm thickness (Gold), h	200	nm
Dielectric spacer thickness, $g_{0}$	200	nm
Insulation layer thickness, $d_{i}$	50	nm
Pull-in voltage, $V_{\mathrm{pull}-\mathrm{in}}$	0.132	V
Resonant frequency, $f_{r}$	45.75	kHz
Tuning range, $C_{\max}/C_{\min}$	1.68:1	-
Zero bias capacitance range	1–2	pF
Capacitance change resolution	1	aF
Substrate	HPFS™	

**Table 4:** Material properties [25].

Property	BCB	Gold	Unit
Young’s modulus, E	2.9	79	GPa
Poisson ratio, v	0.34	0.44	-
Density, $\rho_{m}$	1050	19,300	Kg/m^3^
Relative permittivity, *ε*_r_	2.65	6.9	-

## Data Availability

Not applicable.
